# Bis­(benzimidazole-κ*N* ^3^)bis(2-benzoyl­benzoato-κ*O*)copper(II)

**DOI:** 10.1107/S1600536809017139

**Published:** 2009-05-14

**Authors:** M. Hakkı Yıldırım, Zerrin Heren, Hümeyra Paşaoğlu, Derya Hıra, Orhan Büyükgüngör

**Affiliations:** a Department of Physics, Faculty of Arts and Sciences, Ondokuz Mayıs University, Kurupelit, TR-55139, Samsun, Turkey; b Department of Chemistry, Faculty of Arts and Sciences, Ondokuz Mayıs University, Kurupelit, TR-55139, Samsun, Turkey

## Abstract

In the title centrosymmetric mononuclear copper(II) compound, [Cu(C_14_H_9_O_3_)_2_(C_7_H_6_N_2_)_2_], the central Cu^II^ ion is coordinated by two N atoms from two symmetry-related benzimidazole (bim) ligands and two O atoms from two symmetry-related 2-benzoyl­benzoate (2-byba) ligands in a square-planar geometry. The mol­ecules are linked into chains running along the *b* axis by N—H⋯O hydrogen bonds and the chains are cross-linked by C—H⋯π inter­actions.

## Related literature

For general background to 2-benzoyl­benzoate, see: Diop *et al.* (2006 ▶, 2007 ▶); Foreman *et al.* (2001 ▶); Jones *et al.* (1996 ▶); Martin & Valente (1998 ▶); Prout *et al.* (1996 ▶); Song *et al.* (2005 ▶). For the crystal structure of 2-benzoyl­benzoate, see: Lalancette *et al.* (1990 ▶). For graph-set notation, see: Bernstein *et al.* (1995 ▶).
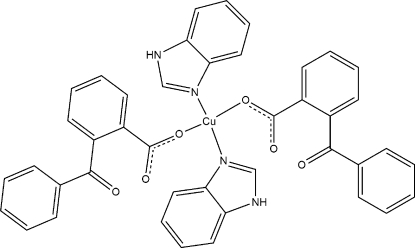


## Experimental

### 

#### Crystal data

[Cu(C_14_H_9_O_3_)_2_(C_7_H_6_N_2_)_2_]
*M* *_r_* = 750.24
Monoclinic, 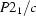

*a* = 10.8692 (6) Å
*b* = 7.4521 (3) Å
*c* = 23.0874 (15) Å
β = 95.111 (5)°
*V* = 1862.61 (18) Å^3^
*Z* = 2
Mo *K*α radiation
μ = 0.64 mm^−1^
*T* = 296 K
0.41 × 0.39 × 0.23 mm

#### Data collection

Stoe IPDS II diffractometer
Absorption correction: integration (*X-RED32*; Stoe & Cie, 2002 ▶) *T*_min_ = 0.550, *T*_max_ = 0.792
10706 measured reflections
3859 independent reflections
2987 reflections with *I* > 2σ(*I*)
*R*_int_ = 0.027

#### Refinement

*R*[*F* ^2^ > 2σ(*F* ^2^)] = 0.036
*wR*(*F* ^2^) = 0.103
*S* = 1.05
3859 reflections
241 parameters
H-atom parameters constrained
Δρ_max_ = 0.19 e Å^−3^
Δρ_min_ = −0.43 e Å^−3^

### 

Data collection: *X-AREA* (Stoe & Cie, 2002 ▶); cell refinement: *X-AREA*; data reduction: *X-RED32* (Stoe & Cie, 2002 ▶); program(s) used to solve structure: *SHELXS97* (Sheldrick, 2008 ▶); program(s) used to refine structure: *SHELXL97* (Sheldrick, 2008 ▶); molecular graphics: *ORTEP-3 for Windows* (Farrugia, 1997 ▶); software used to prepare material for publication: *WinGX* (Farrugia, 1999 ▶).

## Supplementary Material

Crystal structure: contains datablocks global, I. DOI: 10.1107/S1600536809017139/ci2798sup1.cif

Structure factors: contains datablocks I. DOI: 10.1107/S1600536809017139/ci2798Isup2.hkl

Additional supplementary materials:  crystallographic information; 3D view; checkCIF report

## Figures and Tables

**Table 1:** Selected bond lengths (Å)

N1—Cu1	1.9916 (17)
O1—Cu1	1.9474 (13)

**Table 2:** Hydrogen-bond geometry (Å, °)

*D*—H⋯*A*	*D*—H	H⋯*A*	*D*⋯*A*	*D*—H⋯*A*
N2—H2⋯O2^i^	0.86	1.90	2.747 (2)	169 (2)
C5—H5⋯*Cg*1^ii^	0.93	2.67	3.560 (2)	160 (2)
C11—H11⋯*Cg*2^iii^	0.93	2.95	3.760 (2)	146 (2)

Symmetry codes: (i) 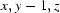; (ii) 
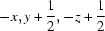
; (iii) 
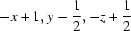
. *Cg*1 and *Cg*2 are centroids of the C9–C14 and N1/C15/N2/C16/C21 rings, respectively.

**Fig. 1.**
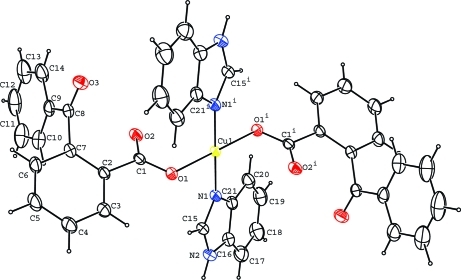
The molecular structure of the title complex, showing the atom-numbering scheme. Displacement ellipsoids are drawn at the 20% probability. Symmetry code: (i) 1 - x, 1 - y, 1 - z.

**Fig. 2.**
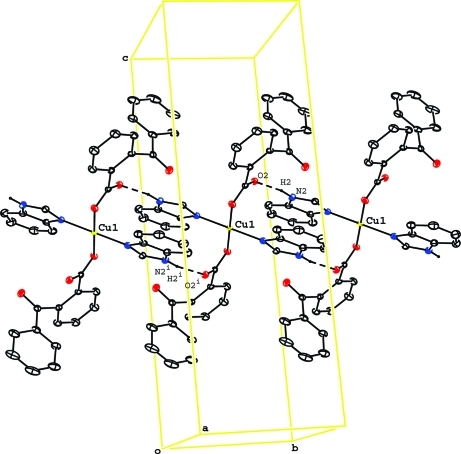
Part of the crystal structure of the title complex showing the chain of R22(16) rings along [010] generated by N—H···O hydrogen bonds (dashed lines). H atoms not involved in hydrogen bonds have been omitted for clarity.

## supplementary crystallographic information

### Comment

Altough the crystal structure of 2-byba was investigated in 1990 (Lalancette *et al.*, 1990), only a limited number of articles have focused on 2-byba complexes. Consequently, this study, among with our ongoing study of characterization of mixed-ligand metal complexes, will provide a new example of copper(II) complexes with 2-byba.

In title compound, the Cu^II^ ion lying on a centre of symmetry, is in a square-planar coordination geometry formed by two symmetry related 2-byba and two symmetry related bim ligands. Both ligands are monodentate with the 2-byba coordinates through carboxylate O atom and bim coordinates through the aromatic N atom.

The molecular packing is mainly stabilized by strong intermolecular N—H···O hydrogen bonds (Table 2 and Fig. 2) and C—H···π interactions. Atom N2 in the molecule at (*x*, *y*, *z*) acts as hydrogen-bond donor, *via* H2, to atom O2 in the molecule at (*x*, *y* - 1, *z*) forming *C*(8) chains with *R*_2_^2^(16) rings (Bernstein *et al.*, 1995). These chains run parallel to the [010] (Fig. 2). The chains are inter-connected to each other by C5—H5···*Cg*1^iii^ and C11—H11···*Cg*2^iv^ interactions along the [001] and [101], respectively. *Cg*1 and *Cg*2 are centroids of the C9–C14 and N1/C15/N2/C16/C21 rings, respectively and symmetry codes (iii) and (iv) are as in Table 2.

### Experimental

For the preparation of the title complex, a solution of cupric acetate (0.18 g, 1 mmol) in methanol (10 ml) was added to a solution of 2-byba (0.45 g, 2 mmol) in methanol (10 ml) and the solution was stirred for 15 min at 333 K. Solution of bim (0.23 g, 2 mmol) in methanol (10 ml) was added to the former solution. Final solution was left to evaporate slowly at room temperature. After one week, violet stick crystals of the title complex were isolated.

### Refinement

All H atoms were placed in calculated positions and constrained to ride on their parents atoms, with C—H = 0.93 Å, N—H = 0.86 Å and *U*_iso_(H) = 1.2*U*_eq_(C,N).

### Crystal data

**Table:**

[Cu(C_14_H_9_O_3_)_2_(C_7_H_6_N_2_)_2_]	*F*(000) = 774
*M**_r_* = 750.24	*D*_x_ = 1.338 Mg m^−^^3^
Monoclinic, *P*2_1_/*c*	Mo *K*α radiation, λ = 0.71073 Å
Hall symbol: -P 2ybc	Cell parameters from 13271 reflections
*a* = 10.8692 (6) Å	θ = 1.8–27.2°
*b* = 7.4521 (3) Å	µ = 0.64 mm^−^^1^
*c* = 23.0874 (15) Å	*T* = 296 K
β = 95.111 (5)°	Block, violet
*V* = 1862.61 (18) Å^3^	0.41 × 0.39 × 0.23 mm
*Z* = 2	

### Data collection

**Table:**

Stoe IPDS II diffractometer	3859 independent reflections
Radiation source: fine-focus sealed tube	2987 reflections with *I* > 2σ(*I*)
graphite	*R*_int_ = 0.027
Detector resolution: 6.67 pixels mm^-1^	θ_max_ = 26.5°, θ_min_ = 1.8°
ω scans	*h* = −13→13
Absorption correction: integration (*X-RED32*; Stoe & Cie, 2002)	*k* = −9→9
*T*_min_ = 0.550, *T*_max_ = 0.792	*l* = −28→28
10706 measured reflections	

### Refinement

**Table:**

Refinement on *F*^2^	Primary atom site location: structure-invariant direct methods
Least-squares matrix: full	Secondary atom site location: difference Fourier map
*R*[*F*^2^ > 2σ(*F*^2^)] = 0.036	Hydrogen site location: inferred from neighbouring sites
*wR*(*F*^2^) = 0.103	H-atom parameters constrained
*S* = 1.05	*w* = 1/[σ^2^(*F*_o_^2^) + (0.063*P*)^2^ + 0.0342*P*] where *P* = (*F*_o_^2^ + 2*F*_c_^2^)/3
3859 reflections	(Δ/σ)_max_ = 0.001
241 parameters	Δρ_max_ = 0.19 e Å^−^^3^
0 restraints	Δρ_min_ = −0.43 e Å^−^^3^

### Special details

**Table:**

Geometry. All e.s.d.'s (except the e.s.d. in the dihedral angle between two l.s. planes) are estimated using the full covariance matrix. The cell e.s.d.'s are taken into account individually in the estimation of e.s.d.'s in distances, angles and torsion angles; correlations between e.s.d.'s in cell parameters are only used when they are defined by crystal symmetry. An approximate (isotropic) treatment of cell e.s.d.'s is used for estimating e.s.d.'s involving l.s. planes.
Refinement. Refinement of *F*^2^ against ALL reflections. The weighted *R*-factor *wR* and goodness of fit *S* are based on *F*^2^, conventional *R*-factors *R* are based on *F*, with *F* set to zero for negative *F*^2^. The threshold expression of *F*^2^ > σ(*F*^2^) is used only for calculating *R*-factors(gt) *etc*. and is not relevant to the choice of reflections for refinement. *R*-factors based on *F*^2^ are statistically about twice as large as those based on *F*, and *R*- factors based on ALL data will be even larger.

### Fractional atomic coordinates and isotropic or equivalent isotropic displacement parameters (Å^2^)

**Table:**

	*x*	*y*	*z*	*U*_iso_*/*U*_eq_	
C1	0.67880 (16)	0.5839 (2)	0.58664 (7)	0.0451 (4)	
C2	0.80566 (16)	0.5833 (3)	0.61777 (7)	0.0463 (4)	
C3	0.8821 (2)	0.4359 (3)	0.61387 (10)	0.0671 (6)	
H3	0.8554	0.3388	0.5907	0.080*	
C4	0.9978 (2)	0.4320 (4)	0.64415 (12)	0.0822 (7)	
H4	1.0478	0.3314	0.6420	0.099*	
C5	1.0388 (2)	0.5765 (4)	0.67729 (11)	0.0784 (7)	
H5	1.1174	0.5750	0.6969	0.094*	
C6	0.96404 (18)	0.7232 (3)	0.68158 (9)	0.0631 (5)	
H6	0.9920	0.8200	0.7045	0.076*	
C7	0.84674 (15)	0.7290 (3)	0.65200 (7)	0.0460 (4)	
C8	0.77333 (16)	0.8979 (3)	0.65665 (8)	0.0512 (4)	
C9	0.71310 (18)	0.9262 (4)	0.71160 (9)	0.0619 (5)	
C10	0.7100 (2)	0.7915 (4)	0.75275 (10)	0.0757 (7)	
H10	0.7439	0.6797	0.7461	0.091*	
C11	0.6561 (3)	0.8237 (6)	0.80394 (12)	0.1086 (12)	
H11	0.6537	0.7334	0.8316	0.130*	
C12	0.6068 (3)	0.9868 (8)	0.81363 (17)	0.130 (2)	
H12	0.5720	1.0072	0.8484	0.156*	
C13	0.6068 (3)	1.1197 (7)	0.77439 (19)	0.1323 (18)	
H13	0.5708	1.2295	0.7819	0.159*	
C14	0.6616 (3)	1.0930 (5)	0.72159 (12)	0.1001 (11)	
H14	0.6631	1.1846	0.6943	0.120*	
C15	0.53325 (19)	0.1542 (3)	0.55656 (8)	0.0569 (5)	
H15	0.6179	0.1741	0.5577	0.068*	
C16	0.3574 (2)	0.0187 (3)	0.56527 (9)	0.0625 (5)	
C17	0.2641 (3)	−0.1000 (4)	0.57604 (11)	0.0856 (8)	
H17	0.2811	−0.2148	0.5904	0.103*	
C18	0.1454 (3)	−0.0395 (5)	0.56439 (13)	0.0947 (9)	
H18	0.0798	−0.1150	0.5707	0.114*	
C19	0.1216 (2)	0.1327 (5)	0.54336 (12)	0.0886 (8)	
H19	0.0398	0.1694	0.5363	0.106*	
C20	0.2139 (2)	0.2516 (4)	0.53244 (10)	0.0698 (6)	
H20	0.1962	0.3665	0.5184	0.084*	
C21	0.33531 (19)	0.1912 (3)	0.54354 (8)	0.0564 (5)	
N1	0.44902 (15)	0.2755 (2)	0.53809 (7)	0.0534 (4)	
N2	0.48416 (18)	0.0022 (2)	0.57316 (8)	0.0630 (4)	
H2	0.5243	−0.0906	0.5865	0.076*	
O1	0.66166 (13)	0.47949 (17)	0.54318 (7)	0.0602 (4)	
O2	0.59825 (11)	0.68310 (18)	0.60404 (6)	0.0527 (3)	
O3	0.77108 (16)	1.0089 (2)	0.61852 (7)	0.0722 (4)	
Cu1	0.5000	0.5000	0.5000	0.04882 (12)	

### Atomic displacement parameters (Å^2^)

**Table:**

	*U*^11^	*U*^22^	*U*^33^	*U*^12^	*U*^13^	*U*^23^
C1	0.0432 (9)	0.0423 (9)	0.0471 (9)	0.0009 (8)	−0.0097 (7)	0.0089 (8)
C2	0.0400 (9)	0.0524 (10)	0.0444 (9)	0.0039 (8)	−0.0074 (7)	0.0045 (8)
C3	0.0575 (12)	0.0659 (13)	0.0735 (13)	0.0167 (11)	−0.0183 (10)	−0.0108 (11)
C4	0.0587 (13)	0.0899 (17)	0.0925 (17)	0.0313 (13)	−0.0228 (12)	−0.0147 (15)
C5	0.0447 (11)	0.1023 (19)	0.0831 (16)	0.0160 (12)	−0.0230 (10)	−0.0076 (15)
C6	0.0468 (10)	0.0767 (15)	0.0623 (12)	0.0013 (10)	−0.0154 (9)	−0.0075 (10)
C7	0.0391 (8)	0.0577 (11)	0.0400 (8)	0.0008 (8)	−0.0026 (7)	0.0034 (8)
C8	0.0439 (9)	0.0549 (12)	0.0517 (10)	−0.0014 (8)	−0.0136 (7)	−0.0025 (9)
C9	0.0412 (9)	0.0826 (14)	0.0587 (11)	0.0084 (10)	−0.0137 (8)	−0.0201 (11)
C10	0.0642 (13)	0.105 (2)	0.0580 (12)	−0.0193 (13)	0.0066 (10)	−0.0134 (13)
C11	0.0814 (19)	0.180 (4)	0.0665 (16)	−0.037 (2)	0.0193 (14)	−0.0270 (19)
C12	0.0559 (15)	0.242 (6)	0.091 (2)	0.008 (2)	−0.0006 (16)	−0.072 (3)
C13	0.081 (2)	0.199 (5)	0.109 (3)	0.072 (3)	−0.0315 (19)	−0.079 (3)
C14	0.0873 (18)	0.120 (3)	0.0855 (18)	0.0499 (18)	−0.0337 (14)	−0.0394 (18)
C15	0.0591 (11)	0.0524 (11)	0.0557 (11)	0.0122 (9)	−0.0141 (9)	0.0009 (9)
C16	0.0701 (13)	0.0646 (14)	0.0509 (11)	0.0033 (11)	−0.0047 (9)	−0.0010 (9)
C17	0.096 (2)	0.085 (2)	0.0740 (15)	−0.0153 (16)	−0.0011 (14)	0.0102 (14)
C18	0.0803 (18)	0.121 (3)	0.0814 (18)	−0.0209 (18)	0.0020 (15)	0.0108 (17)
C19	0.0579 (13)	0.133 (3)	0.0741 (15)	0.0003 (16)	0.0015 (11)	−0.0062 (17)
C20	0.0598 (13)	0.0883 (16)	0.0596 (12)	0.0155 (12)	−0.0038 (10)	−0.0032 (11)
C21	0.0579 (11)	0.0641 (12)	0.0452 (10)	0.0074 (10)	−0.0061 (8)	−0.0054 (8)
N1	0.0555 (9)	0.0508 (9)	0.0507 (8)	0.0136 (8)	−0.0126 (7)	−0.0029 (7)
N2	0.0714 (11)	0.0535 (10)	0.0612 (10)	0.0123 (9)	−0.0106 (9)	0.0077 (8)
O1	0.0585 (8)	0.0495 (8)	0.0668 (8)	0.0097 (6)	−0.0270 (7)	−0.0065 (6)
O2	0.0412 (6)	0.0570 (8)	0.0582 (7)	0.0047 (6)	−0.0037 (6)	0.0086 (6)
O3	0.0774 (10)	0.0606 (9)	0.0751 (10)	−0.0004 (8)	−0.0123 (8)	0.0153 (8)
Cu1	0.05087 (19)	0.04200 (18)	0.04936 (19)	0.01286 (14)	−0.01906 (13)	−0.00646 (13)

### Geometric parameters (Å, °)

**Table:**

C1—O2	1.240 (2)	C13—C14	1.418 (5)
C1—O1	1.270 (2)	C13—H13	0.93
C1—C2	1.497 (2)	C14—H14	0.93
C2—C3	1.385 (3)	C15—N2	1.323 (3)
C2—C7	1.393 (3)	C15—N1	1.329 (2)
C3—C4	1.384 (3)	C15—H15	0.93
C3—H3	0.93	C16—N2	1.379 (3)
C4—C5	1.373 (4)	C16—C17	1.384 (4)
C4—H4	0.93	C16—C21	1.393 (3)
C5—C6	1.371 (3)	C17—C18	1.371 (4)
C5—H5	0.93	C17—H17	0.93
C6—C7	1.392 (3)	C18—C19	1.388 (5)
C6—H6	0.93	C18—H18	0.93
C7—C8	1.499 (3)	C19—C20	1.379 (4)
C8—O3	1.207 (2)	C19—H19	0.93
C8—C9	1.494 (3)	C20—C21	1.396 (3)
C9—C10	1.385 (4)	C20—H20	0.93
C9—C14	1.390 (4)	C21—N1	1.402 (3)
C10—C11	1.386 (4)	N1—Cu1	1.9916 (17)
C10—H10	0.93	N2—H2	0.86
C11—C12	1.355 (6)	O1—Cu1	1.9474 (13)
C11—H11	0.93	Cu1—O1^i^	1.9474 (13)
C12—C13	1.342 (6)	Cu1—N1^i^	1.9916 (17)
C12—H12	0.93		
			
O2—C1—O1	124.26 (16)	C14—C13—H13	119.9
O2—C1—C2	119.54 (16)	C9—C14—C13	118.3 (4)
O1—C1—C2	116.20 (16)	C9—C14—H14	120.8
C3—C2—C7	119.45 (17)	C13—C14—H14	120.8
C3—C2—C1	120.21 (18)	N2—C15—N1	113.00 (19)
C7—C2—C1	120.32 (16)	N2—C15—H15	123.5
C4—C3—C2	120.5 (2)	N1—C15—H15	123.5
C4—C3—H3	119.8	N2—C16—C17	131.2 (2)
C2—C3—H3	119.8	N2—C16—C21	105.5 (2)
C5—C4—C3	120.0 (2)	C17—C16—C21	123.3 (2)
C5—C4—H4	120.0	C18—C17—C16	116.5 (3)
C3—C4—H4	120.0	C18—C17—H17	121.7
C6—C5—C4	120.1 (2)	C16—C17—H17	121.7
C6—C5—H5	120.0	C17—C18—C19	121.0 (3)
C4—C5—H5	120.0	C17—C18—H18	119.5
C5—C6—C7	120.8 (2)	C19—C18—H18	119.5
C5—C6—H6	119.6	C20—C19—C18	122.8 (3)
C7—C6—H6	119.6	C20—C19—H19	118.6
C6—C7—C2	119.15 (18)	C18—C19—H19	118.6
C6—C7—C8	117.45 (18)	C19—C20—C21	116.8 (3)
C2—C7—C8	123.33 (15)	C19—C20—H20	121.6
O3—C8—C9	122.7 (2)	C21—C20—H20	121.6
O3—C8—C7	119.97 (18)	C16—C21—C20	119.5 (2)
C9—C8—C7	117.12 (18)	C16—C21—N1	108.72 (18)
C10—C9—C14	119.9 (2)	C20—C21—N1	131.7 (2)
C10—C9—C8	121.3 (2)	C15—N1—C21	104.72 (17)
C14—C9—C8	118.7 (3)	C15—N1—Cu1	120.13 (15)
C9—C10—C11	119.9 (3)	C21—N1—Cu1	134.28 (13)
C9—C10—H10	120.1	C15—N2—C16	108.04 (17)
C11—C10—H10	120.1	C15—N2—H2	126.0
C12—C11—C10	120.0 (4)	C16—N2—H2	126.0
C12—C11—H11	120.0	C1—O1—Cu1	114.74 (12)
C10—C11—H11	120.0	O1—Cu1—O1^i^	180.0
C13—C12—C11	121.8 (3)	O1—Cu1—N1^i^	91.04 (6)
C13—C12—H12	119.1	O1^i^—Cu1—N1^i^	88.96 (6)
C11—C12—H12	119.1	O1—Cu1—N1	88.96 (6)
C12—C13—C14	120.1 (4)	O1^i^—Cu1—N1	91.04 (6)
C12—C13—H13	119.9	N1^i^—Cu1—N1	180.00 (5)
			
O2—C1—C2—C3	156.78 (19)	C12—C13—C14—C9	0.8 (5)
O1—C1—C2—C3	−23.2 (3)	N2—C16—C17—C18	178.5 (3)
O2—C1—C2—C7	−21.7 (2)	C21—C16—C17—C18	−0.3 (4)
O1—C1—C2—C7	158.32 (17)	C16—C17—C18—C19	−0.5 (4)
C7—C2—C3—C4	0.7 (3)	C17—C18—C19—C20	0.6 (5)
C1—C2—C3—C4	−177.8 (2)	C18—C19—C20—C21	0.0 (4)
C2—C3—C4—C5	−1.5 (4)	N2—C16—C21—C20	−178.15 (19)
C3—C4—C5—C6	1.6 (4)	C17—C16—C21—C20	0.9 (3)
C4—C5—C6—C7	−0.8 (4)	N2—C16—C21—N1	0.6 (2)
C5—C6—C7—C2	0.1 (3)	C17—C16—C21—N1	179.7 (2)
C5—C6—C7—C8	−177.1 (2)	C19—C20—C21—C16	−0.8 (3)
C3—C2—C7—C6	0.0 (3)	C19—C20—C21—N1	−179.2 (2)
C1—C2—C7—C6	178.49 (17)	N2—C15—N1—C21	−0.1 (2)
C3—C2—C7—C8	176.95 (18)	N2—C15—N1—Cu1	−170.88 (13)
C1—C2—C7—C8	−4.6 (3)	C16—C21—N1—C15	−0.3 (2)
C6—C7—C8—O3	98.0 (2)	C20—C21—N1—C15	178.2 (2)
C2—C7—C8—O3	−79.0 (2)	C16—C21—N1—Cu1	168.53 (14)
C6—C7—C8—C9	−77.5 (2)	C20—C21—N1—Cu1	−12.9 (3)
C2—C7—C8—C9	105.5 (2)	N1—C15—N2—C16	0.5 (2)
O3—C8—C9—C10	175.5 (2)	C17—C16—N2—C15	−179.6 (2)
C7—C8—C9—C10	−9.2 (3)	C21—C16—N2—C15	−0.7 (2)
O3—C8—C9—C14	−5.8 (3)	O2—C1—O1—Cu1	5.1 (2)
C7—C8—C9—C14	169.59 (19)	C2—C1—O1—Cu1	−174.87 (11)
C14—C9—C10—C11	−0.3 (4)	C1—O1—Cu1—N1^i^	77.26 (14)
C8—C9—C10—C11	178.4 (2)	C1—O1—Cu1—N1	−102.74 (14)
C9—C10—C11—C12	−0.1 (4)	C15—N1—Cu1—O1	−26.68 (15)
C10—C11—C12—C13	1.0 (5)	C21—N1—Cu1—O1	165.80 (18)
C11—C12—C13—C14	−1.3 (6)	C15—N1—Cu1—O1^i^	153.32 (15)
C10—C9—C14—C13	0.0 (4)	C21—N1—Cu1—O1^i^	−14.20 (18)
C8—C9—C14—C13	−178.8 (2)		

Symmetry codes: (i) −*x*+1, −*y*+1, −*z*+1.

### Hydrogen-bond geometry (Å, °)

**Table:**

*D*—H···*A*	*D*—H	H···*A*	*D*···*A*	*D*—H···*A*
N2—H2···O2^ii^	0.86	1.90	2.747 (2)	169 (2)
C5—H5···Cg1^iii^	0.93	2.67	3.560 (2)	160 (2)
C11—H11···Cg2^iv^	0.93	2.95	3.760 (2)	146 (2)

Symmetry codes: (ii) *x*, *y*−1, *z*; (iii) −*x*, *y*+1/2, −*z*+1/2; (iv) −*x*+1, *y*−1/2, −*z*+1/2.
